# Systems Biology Integration and Screening of Reliable Prognostic Markers to Create Synergies in the Control of Lung Cancer Patients

**DOI:** 10.3389/fmolb.2020.00047

**Published:** 2020-04-07

**Authors:** Aman Chandra Kaushik, Aamir Mehmood, Dong-Qing Wei, Xiaofeng Dai

**Affiliations:** ^1^Wuxi School of Medicine, Jiangnan University, Wuxi, China; ^2^School of Life Sciences and Biotechnology, Shanghai Jiao Tong University, Shanghai, China

**Keywords:** lung cancer, TCGA, survival, systems biology, prognostic biomarkers

## Abstract

This study aims to achieve a clearer and stronger understanding of all the mechanisms involved in the occurrence as well as in the progression of lung cancer along with discovering trustworthy prognostic markers. We combined four gene expression profiles (GSE19188, GSE19804, GSE101929, and GSE18842) from the GEO database and screened the commonly differentially expressed genes (CDEGs). We performed differentially expressed group analysis on CDEGs, alteration and mutational analysis, and expression level verification of core differential genes. Systems biology discoveries in our examination are predictable with past reports. Curiously, our examination revealed that screened biomarker adjustments, for the most part, coexist in lung cancer. After screening 952 CDEGs, we found that the up-regulation of neuromedin U (NMU) and GTSE1 in the case of lung cancer is related to poor prognosis. On the other hand, FOS CDKN1C expression is associated with poor prognosis and is responsible for the down-regulation of CDKN1C and FOS. Changes in these qualities are on free pathways to lung cancer and are not usually of combined quality variety. Even though biomarkers were related to both survival occasions in our examination, it gives us another point of view while playing out the investigation of hereditary changes and clinical highlights employing information mining. Based on our results, we found potential and prospective clinical applications in GTSE1, NMU, FOS, and CDKN1C to act as prognostic markers in case of lung cancer.

## Graphical Abstract

This picture depicts the whole methodology’s pipeline used for the current work that involves genomics and systems biology to scan for potential lung cancer biomarkers.

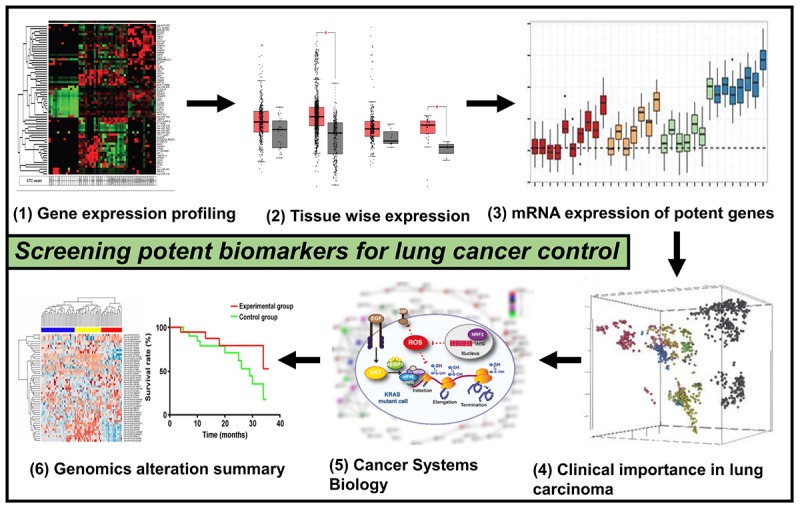

## Introduction

Lung cancer cases are among the most reported tumors that have peak sickness and impermanence rates worldwide (Siegel et al., 2019). Various factors could result in the development of such condition; however, smoking, which may be regular or passive, radon gas, asbestos fibers, familial predisposition, lung diseases, and air pollution is the main cause. The symptoms may vary from person to person and case to case, but some regular signs involve ongoing cough, blood-streaked saliva, puffed hoarseness, or some slight infection that keeps on coming. As per the various diagnostic types, lung cancer is majorly grouped into two types, namely, small cell lung cancer (SCLC) and non-small cell lung cancer (NSCLC), of which nearly 90% of the cases account for NSCLC (Siegel et al., 2018). Although a lot of advancement has taken place in the field of science and technology and medical methods, the 5-year existence percentage of patients who have SCLC continues to be merely 30% due to factors such as tumor recurrence and metastasis. Finding tumor markers with accurate prognosis can further aid in understanding the direction and mechanism of tumor progression and provide patients with personalized treatment plans to advance the overall endurance of sufferers.

In the present scenario, the integration of high-throughput omics technology and bioinformatics analysis continues to be a significant and effective research method in clinical research to discover target molecules associated with diseases. Moreover, it is considered to be a reliable technique for bioinformatics analysis of the integration of a huge quantity of omics data to discover targets that have potential application importance, for instance, researches on colorectal cancer (Luca et al., 2019), oral cancer (Di et al., 2019; Pan et al., 2019), ovarian cancer (Hu et al., 2019), osteosarcoma (Ma et al., 2019), and lung cancer (Feng et al., 2019). In the present study, the first step we did was to collect expression profiles of NSCLC mRNA from the GEO database and inspected them for genes that are commonly differentially expressed. The systems biology workbench was used to execute analysis of gene network and visual analysis of network on genes that are commonly differentially expressed, and after this, we chose the major differentially expressed genes from commonly differentially expressed genes Common differentially expressed genes (cDEGs). The prognostic value of major differential genes in NSCLC patients was then analyzed based on metainspection (Graphical Abstract).

## Materials and Methods

### Data Retrieval and Acquisition

The gene’s countenance contours of [GSE19188 (Hou et al., 2010), GSE19804 (Lu et al., 2010), GSE101929 (Mitchell et al., 2017), and GSE18842 (Sanchez-Palencia et al., 2011)] were acquired from GEO database. All the microarray data belonging to GSE19188, GSE19804, GSE101929, and GSE18842 exist on GPL570 Platforms (Affymetrix Human Genome U133 Plus 2.0 Array), which was inclusive of 54 NSCLC tissues and 49 normal lung matching tissues, 60 tissues of NSCLC and 60 normal lung matching tissues, 30 tissues of NSCLC and 34 normal lung matching tissues, and 46 tissues of NSCLC and 45 normal lung matching tissues, respectively. We used (Teng et al., 2016) R package to plot the gene expression (transcript per million) on the basis of gene length for normalization, where total reads were mapped to gene × 10^3^/gene length in base pairs (bp) shown in Figure 2.

### mRNA Expression Profiling

The microarrays expression in lung tissues was used to identify those genes that are differentially expressed (DEGs). The lung tissues used in this case were both of the tumor and coordinated head-to-head non-cancerous. In order to scan for genes associated with cancer, detailed literature review was considered, integrating bioinformatics approaches. The GTSE1, neuromedin U (NMU), FOS, and CDKN1C were obtained for confirming the aspirant’s gene transcription and expression degree. In the end, Fisher’s test was conducted to analyze the connection among the pathological characteristics and aspirant genes.

### Functional Identification of GTSE1, NMU, FOS, and CDKN1C Using Systems Biology Approach

To plan and perform the GTSE1, NMU, FOS, and CDKN1C and their associated genes in biological mechanism, the computational systems biology workbench was employed. The required data for both the direct and indirect linkages were collected by conducting a detailed survey of the available information. A complete biological pathway is formed showing all the interacting species. In this constructed pathway, the entities are signified by nodes, while the edges represent the linkage in between a pair of nodes that reveals their close association. A particular concentration was assigned for the time course simulation of the biochemical pathway that was noted from previous reports. Differentially expressed genes of each and every series were taken for analyses, where criterion was set to adjusted *P* < 0.01 and | logFC| > 1. The analysis began by first screening the DEGs present in each of the dataset with standard *P* < 0.01.

### Kaplan–Meier Survival Breakdown

The analysis of prognostic value of CDEGs in the patients who were suffering from lung cancer was done by Kaplan–Meier, with 54,000 genes in 21 tumors. In this study, we used information on lung cancer from the database to analyze the prognostic value, inclusive of 675 squamous cell carcinomas and 866 adenocarcinomas.

### Validation of CDEGs Expression Levels and Correlation Analysis

For the screening of promising CDEGs, we verified their countenance points in 969 lung cancer models and 735 paracancerous samples where cutoff values were set as | logFC| > 1 and *P* < 0.01. In addition, the correlation between countenance levels and clinical stage of tumors was assessed as well, investigating whether valuable CDEGs are independent influencing factors influencing the prognosis of lung cancer.

## Results

### Genomic Landscape of GTSE1, NMU, FOS, and CDKN1C in Prognosis of Patients Suffering From Lung Cancer

**FIGURE 1:**
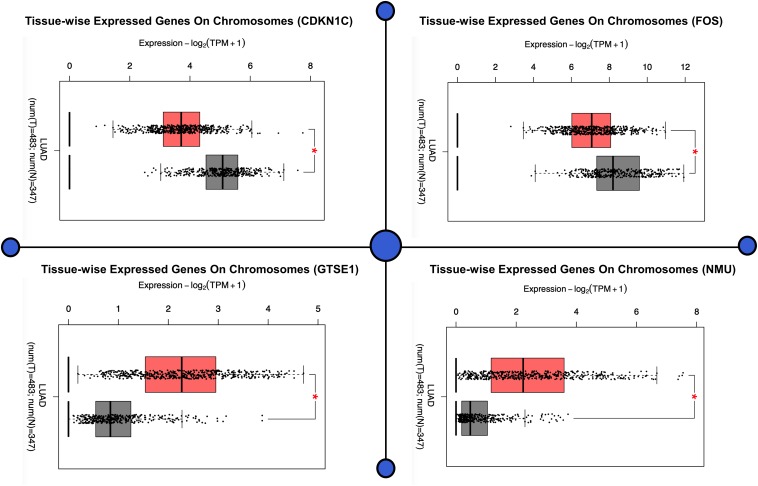
The demonstration of the potential medical value of GTSE1, NMU, FOS, and CDKN1C in the prognosis of patients suffering from lung cancer. This figure shows the difference in the expression pattern on chromosomes of all the four genes in a tissue (tissue-wise expressed genes on chromosomes). The figure demonstrates that four CDEGs were wholly coherent with the results obtained in all the four sets of data, which further demonstrates that the prognostic value of these four targets for lung cancer is good. As per the above results, FOS, GTSE1, CDKN1C, and NMU have good prognostic values for patients with lung cancer, as shown in the figure.

The demonstration of the potential medical value of GTSE1, NMU, FOS, and CDKN1C in the prognosis of patients suffering from lung cancer was done by us in order to verify the levels of expression of FOS, CDKN1C, NMU, and GTSE1. The results illustrated that expression levels of GTSE1 (*P* < 0.05) and NMU (*P* < 0.05) were notably up-regulated, and expression levels of CDKN1C (*P* < 0.05) and FOS (*P* < 0.05) were notably down-regulated in lung adenocarcinoma (LUAD), as well as in lung squamous cell carcinoma, and significantly statistically significant. The results of verification demonstrated that four CDEGs were wholly coherent with the results obtained in all the four sets of data, which further demonstrate that the prognostic value of these four targets for lung cancer is good. As per the above results, FOS, GTSE1, CDKN1C, and NMU have good prognostic values for patients with lung cancer, as shown in Figure 1.

### Expression of CDEGs

**FIGURE 2:**
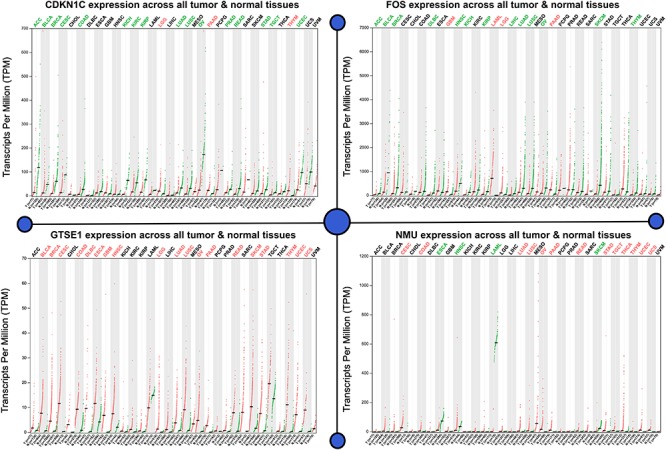
Expression analysis of potent biomarkers. Differential expression screening on four datasets of lung cancer GSE101929, GSE18842, GSE19188, and GSE19804. Gene expression (transcript per million) on the basis of gene length for normalization, where total reads were mapped to gene × 10^3^/gene length in bp. The level of expression accords that all the considered tumor samples are given for each up-regulated and down-regulated genes where the CDKN1C is considered to be the least expressed, whereas FOS is observed to be highly overexpressed.

We executed differential expression screening on four datasets of lung cancer (GSE101929, GSE18842, GSE19188, and GSE19804), which we collected from the GEO database. These datasets consist of 3,179, 3,162, 2,601, and 1,404 genes, which are differentially expressed, of which 952 genes were CDEGs, inclusive of 256 up-regulated genes and 696 down-regulated genes shown in Figure 2.

### mRNA Expression Profiling

**FIGURE 3:**
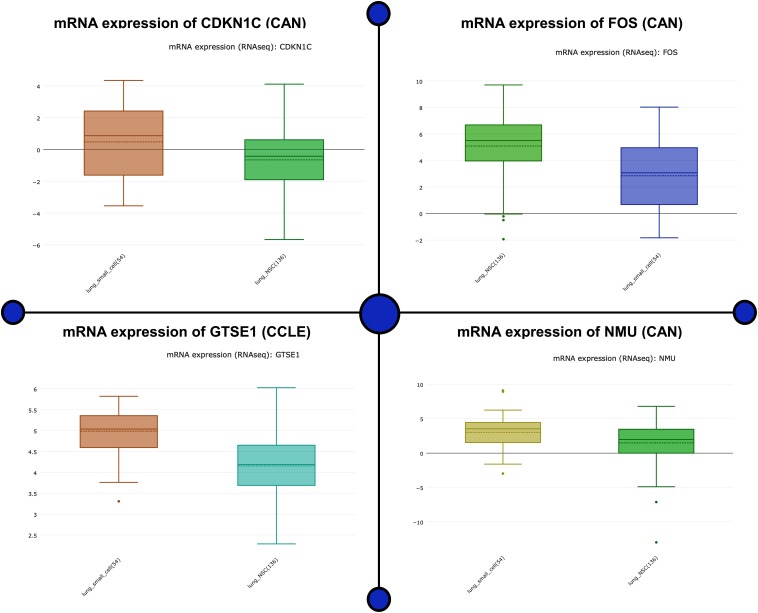
mRNA expression microarrays showed FOS, GTSE1, CDKN1C, and NMU are greatly harbored in lung cancer patients and are observed to be differentially expressed (fold change ≥2.0) in these genes. The up- and down-regulated genes as a potential biomarker are analyzed for its mRNA expression. This figure depicts the mRNA biological impact of this gene by virtue of its intrinsic regulatory nature in patients.

**FIGURE 4:**
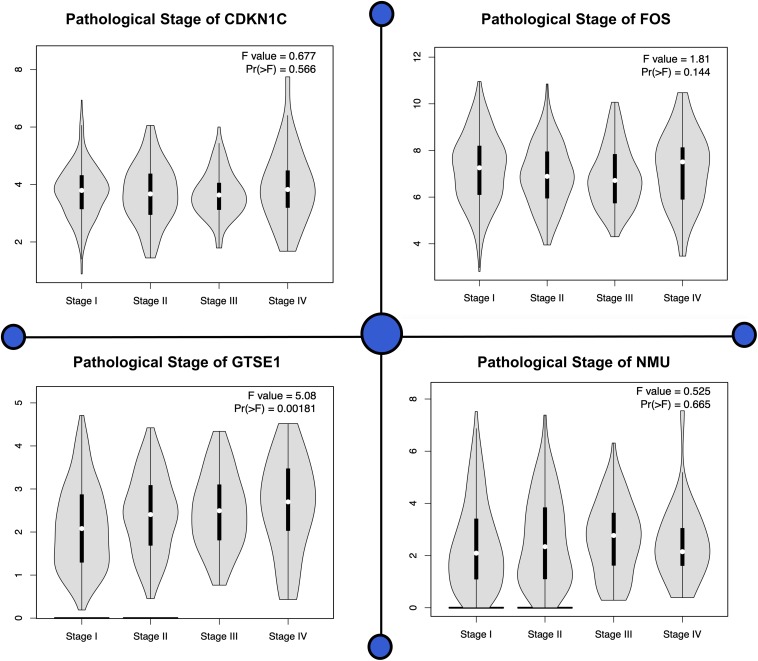
Various pathological stages of all four genes are shown in this plot. For each individual biomarker, four stages can be observed showing significant difference present among all the genes.

Analyzing the mRNA expression microarrays showed FOS, GTSE1, CDKN1C, and NMU are greatly harbored by the patients of lung cancer and are observed to be differentially expressed (fold change ≥2.0) of these genes. Of these differentially expressed qualities, GTSE1 and NMU were overexpressed, whereas the down-regulated qualities were CDKN1C and FOS. The computational techniques used here revealed that CDKN1C and FOS, which are down-regulated genes in lung cancer-positive patients, are closely associated with the compulsive situation and are responsible for the regulation number of biological comebacks. Information assessment regarding CDKN1C and FOS exposed its down-regulation as a separate countenance outline in lung cancer happening in other geographical locations shown in Figure 3. The association breakdown of experimental features disclosed down-regulation of CDKN1C and FOS and its correlation with the development of the diseased condition. In the end, Fisher’s test was conducted to analyze the connection between the pathological characteristics and aspirant genes shown in Figure 4.

### Functional Identification of DEGs Using Systems Biology Approach

**FIGURE 5:**
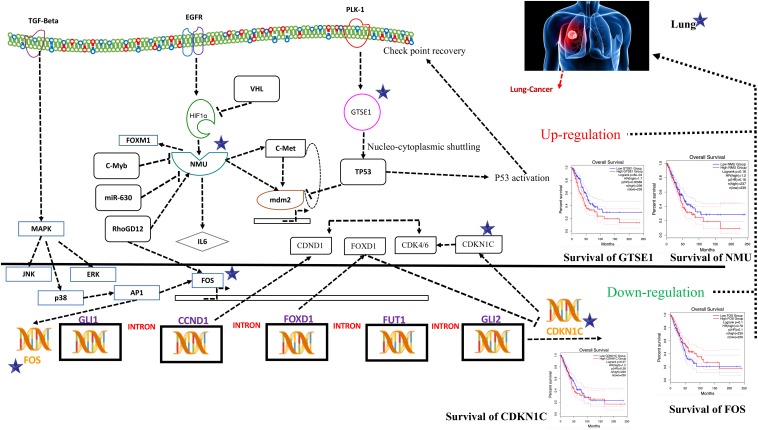
Systems biology is an interdisciplinary field that involves computational and mathematical investigations for modeling a complex biological mechanism. This figure is a depiction of the genes in a lung cancer biochemical pathway and how they interact in a system. FOS, GTSE1, CDKN1C, and NMU could be the potential genes for further evaluation in this regard and may play an important role as biomarkers of lung cancer diagnosis. The CDKN1C, which is also known as p57^kip2^, is claimed to be a tumor suppressor considered to be a potential tumor suppressor caught up in lung cancer in humans. However, recent reports demonstrate CDKN1C in lung cancer is observed to be intensely down-regulated. The low expression of CDKN1C is expressively connected with overall poor survival rate shown in Figure 5. In contrast, the growth-encouraging movement and augmented mobilization of cells are conferred by inducing an exogenous expression of NMU shown in Figure 5. The GTSE1 gene is claimed to be involved in lung cancerous pathways because of its overexpression and negative regulation of p53 expression. It is also observed that its expression is linked with venous invasion, global short survival, and size of the tumor. FOS can be utilized as markers of poor prognosis of lung cancer shown in Figures 5, 6 as pathway for each species.

Systems biology is an interdisciplinary field that involves computational and mathematical investigations for modeling a complex biological mechanism. It mainly addresses the linkages formed within the living systems and tracks changes upon the incorporation of any non-native event using an all-inclusive technique. Narrowing down to cancer systems biology, it mainly involves the use of systems biology’s technology in cancer research, for the sake of examining an ailment as a challenging adaptive system having evolved characteristics at various biological parameters, as shown in Figure 5.

To obtain the synopsis of the role and contribution of 952 CDEGs in the enhancement of lung cancer, we observed that biological processes significantly related to the advancement of lung cancer, angiogenesis, cells’ outside medium association, collagen catabolic progression, and positive regulation of angiogenesis. Moreover, cell components such as cells’ outside medium, protein-rich medium surrounding the cell, and cells’ outer area; collagen trimer and extracellular region; molecular function; integrin binding; and protein-binding and heparin-binding activities of metalloendopeptidase were also found to be tightly linked with the progression of the lung cancer. The results obtained from the signaling pathways and cell adhesion molecules (CAMs) were observed to be most closely related to the occurrence of lung cancer. The ratio of DEGs in lung cancer patients was high and was observed to be responsible for the loss of function in many biological mechanisms. Therefore, it is initially proposed that FOS, GTSE1, CDKN1C, and NMU could be the potential genes for further evaluation in this regard and may play an important role as biomarkers of lung cancer diagnosis, as shown in Figure 5.

The CDKN1C, which is also known as p57^kip2^, is claimed to be a tumor suppressor considered to be a potential tumor suppressor caught up in several types of cancer in humans. However, recent reports (Qiu et al., 2018) demonstrate CDKN1C in breast cancer is observed to be intensely down-regulated equated with normal tissue. Furthermore, the CDKN1C expression is detected to be associated with age and tumor size in The Cancer Genome Atlas (TCGA) cohort containing 708 cases of breast cancer. The low expression of CDKN1C is expressively connected with overall poor survival rate, as shown in Figure 5.

On the other hand, reports on the FOS maintained that its down-regulation might be associated with the pathogenesis of lung cancer (Mahner et al., 2008). This gene and TP53 play as transcription factors and also as target genes in this system, having the ability to self-regulate. The need for transcription factor for TP53 is fulfilled by the FOS (Levin et al., 1995), as shown in Figure 5.

An ample amount of expression is detected regarding NMU in the vast majority of lung cancers. Various analyses have unveiled a substantial connotation of NMU expression with a minor prognosis of patients suffering from NSCLC. The expression of this gene can be suppressed when short interfering RNAs are cast off to treat NSCLC that retards the cell’s development. In contrast, the growth-encouraging movement and augmented mobilization of cells are conferred by inducing an exogenous expression of NMU shown in Figure 5.

**FIGURE 6:**
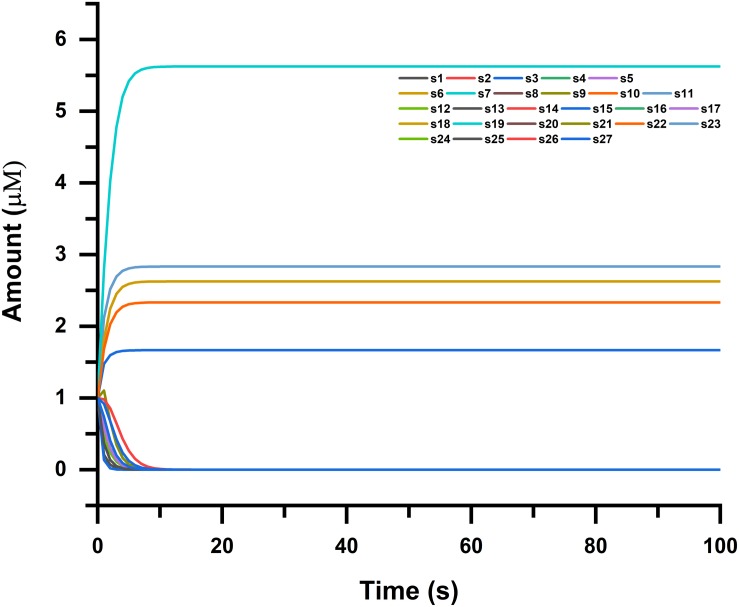
Time course simulation of NMU-, GTSE1-, CDKN1C-, and FOS-associated pathways, where simulation was done in four phases for each species (genes and proteins in the pathway). Narrowing down to cancer systems biology, it mainly involves the use of systems biology’s technology in cancer research, for the sake of examining an ailment as a challenging adaptive system having evolved characteristics at various biological parameters shown in the figure.

The GTSE1 gene is claimed to be involved in many cancerous pathways due to its overexpression and negative regulation p53 expression. Recent reports also claimed that this gene both at its mRNA and protein levels is extremely up-regulated in hepatocellular carcinoma specimens (silencing GTSE-1 expression inhibits proliferation and invasion of hepatocellular carcinoma cells). It is also observed that its expression is linked with venous invasion, global short survival, and size of the tumor shown in Figure 5. The time course simulation’s illustrate elevated NMU and GTSE1 countenance, reduced expression of CDKN1C and FOS, can be utilized as markers of poor prognosis of lung cancer, as shown in Figures 5, 6 showing pathway for each species.

### Survival Analysis

**FIGURE 7:**
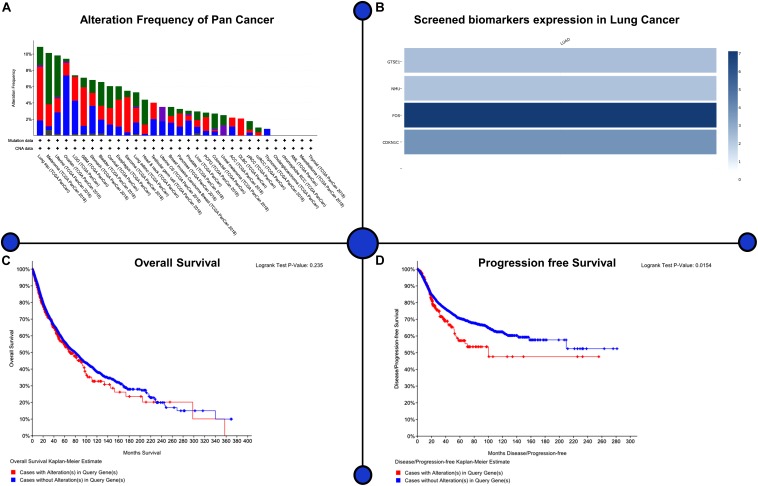
Assessment of prognostic value belonging to GTSE1, NMU, FOS, and CDKN1C in patients with NSCLC. The Kaplan–Meier survival curves of LUAD and their major significance have been drawn to explore their effects on prognosis. Patients with LUAD showed significantly worse prognosis than patients without biomarkers. Lung adenocarcinoma showed worse prognosis than patients with four biomarkers. **(A)** Alteration frequency of pan-cancer, **(B)** screened biomarker expression in lung cancer, **(C)** overall survival, **(D)** progression-free survival.

We selected four major genes, of which GTSE1 (logFC = 1.32, adjusted *P* < 0.001) and NMU (logFC = 2.81, adjusted *P* < 0.001) were found to be up-regulated in the tissues of those patients who had lung cancer, and expression levels of FOS (logFC = -2.22, adjusted *P* < 0.001) and CDKN1C (logFC = -1.56, adjusted *P* < 0.001) were found to be down-regulated. For the assessment of prognostic value belonging to GTSE1, NMU, FOS, and CDKN1C in patients with NSCLC, we analyzed 1,926 NSCLC cases from TCGA, GEO, and EGA databases. It was illustrated through the results that high levels of expression of GTSE1 (*P* < 0.01) and NMU (*P* < 0.01) were very closely associated with shorter complete survival of NSCLC patients, with statistical importance. On the contrary, high levels of expression of CDKN1C [*P* < 0.01 and FOS (*P* < 0.01)] were significantly related to longer survival in patients with NSCLC; these findings illustrate elevated NMU and GTSE1 countenance and reduced expression of CDKN1C and FOS can be utilized as markers of poor prognosis of lung cancer as shown in Figure 7.

## Discussion

The mortality rate of lung cancer is high because it easily metastasizes and lapses in between treatments. Hence, it is of utmost importance to overcome the medical obstruction by accurate prediction of potential prognostic markers of the status of tumor progression. Transcription omics that have high-throughput benefits can be of great help for those who are in medical research to facilitate the screening of the target molecules. So, we combined four mRNA expression profiles belonging to lung cancer.

By carrying out a comparison between NSCLC tissues and paired paracancerous tissues, 952 CDEGs were screened from the four expression profiles, among which had up-regulated expression and 696 CDEGs had down-regulated expression, respectively. It was seen by performing Gene Ontology analysis that CDEGs were majorly supplemented in biological processes such as cell adhering, as well as positive modulation of angiogenesis, and KEGG pathways, such as ECM–receptor interaction and CAMs. In the same way, this result was also reported by Piao et al. (2018).

GTSE1 has been found to be highly expressed in the tumors such as melanoma and lung cancer and is related to the weak prognosis of the patients (Wu et al., 2017; Xu et al., 2018). Additionally, GTSE1 might be participating in tumorigenesis and progression by modulating p53 phosphorylation (Liu et al., 2010, 2019). Neuromedin U is very well known for its uterine smooth muscle contraction inducer. In the meantime, it also contributes to the process of formation and enlargement of various kinds of tumors. For instance, it was reported by Takahashi et al. (2006) that the positive rate of NMU in NSCLC and SCLC was as high as 68 and 82%, and the overexpression of NMU was validated at the transcriptional level and protein level (Takahashi et al., 2006). Moreover, studies have demonstrated that overexpression of NMU is also produced in HER2-overexpressing breast cancer, and overexpression of NMU in breast cancer is linked with reduced prognosis in sufferers (Shetzline et al., 2004; Wu et al., 2007). Similar reports have been reported in the study of clear cell renal cell carcinoma and endometrial carcinoma (Ketterer et al., 2009; Przygodzka et al., 2016; Zhang et al., 2019). CDKN1C is a cancerous lump restrainer gene, which is down-regulated in studies related to gastric cancer (Shin et al., 2000), bladder cancer (Oya and Schulz, 2000), pancreatic cancer (Sato et al., 2005), lung cancer (Sun et al., 2017), and breast cancer (Qiu et al., 2018), and low expression points are connected with reduced prediction in sufferers. Importantly, all of the above research results strongly support our analysis results. Additionally, CDKN1C, GTSE1, NMU, and FOS are not correlated with one another, which indicates that every target can individually be cast off as a predictive marker for lung cancer. Ultimately, the aforementioned studies show the capability of FOS, CDKN1C, GTSE1, and NMU as prognostic markers of lung cancer.

## Conclusion

The molecular specification of lung cancer has considerably changed the categorization and treatment of tumors, becoming a crucial component of diagnosis and oncologic therapy. We executed differential analysis of samples of lung cancer and matching tissues of paracancer and noticed four core CDEGs could be utilized as prognostic markers of lung cancer via correlation analysis and expression level verification. We strongly believe that GTSE1, NMU, FOS, and CDKN1C have potential and clinical application values to act as prognostic markers of lung cancer.

## Data Availability Statement

The raw data supporting the conclusions of this article will be made available by the authors, without undue reservation, to any qualified researcher.

## Author Contributions

AK and D-QW designed the experiments. D-QW, AK, and XD, performed the entire computational experiments and assisted in writing the manuscript. XD, AK analyzed the data and wrote the manuscript. AK, D-QW, XD, and AM read the manuscript and advised on method development. All authors have given approval to the final version of the manuscript.

## Conflict of Interest

The authors declare that the research was conducted in the absence of any commercial or financial relationships that could be construed as a potential conflict of interest.
